# Photobreeding Method for Direct Construction and Continuous Tuning of Strong Metal–Support Interactions at Room Temperature

**DOI:** 10.1002/advs.202509904

**Published:** 2025-09-09

**Authors:** Wei‐Qiang Huang, Ping Bai, Kai‐Bin Jiang, Guo‐Cong Guo

**Affiliations:** ^1^ State Key Laboratory of Structural Chemistry Fujian Institute of Research on the Structure of Matter Chinese Academy of Sciences Fuzhou Fujian 350108 P. R. China; ^2^ Fujian Provincial Key Laboratory of Advanced Inorganic Oxygenated Materials College of Chemistry Fuzhou University Fuzhou Fujian 350108 P. R. China; ^3^ Fujian Science&Technology Innovation Laboratory for Optoelectronic Information of China Fuzhou Fujian 350108 P. R. China

**Keywords:** metal‐support interactions, photochromism, photocatalysis

## Abstract

The construction of strong metal–support interactions (SMSI) is an effective strategy to enhance and control heterogeneous catalysts. However, conventional methods require pre‐synthesized metal‐loaded catalysts, followed by SMSI formation via high‐temperature treatment under oxidative/reductive atmospheres, adsorbate‐mediated treatment, and photo‐treatment, adding complexity to catalyst synthesis and hindering continuous interfacial tuning. In this work, a “photobreeding” method is employed to treat Zn_0.8_Cd_0.2_S, leveraging the UV‐induced photochromic reaction of ZnS to generate metallic Zn at room temperature, while CdS remains inert. Zn_0.8_Cd_0.2_S‐derived CdS directly encapsulates the generated Zn, enabling direct synthesis of SMSI catalysts without additional treatments. Furthermore, the interfacial properties can be continuously tuned by adjusting the photobreeding duration. The catalytic performance of SMSI catalysts synthesized via photobreeding exhibits a 3.1‐fold enhancement over the initial catalysts. To further boost performance, viologen, an electron mediator, is introduced to form a surface charge transfer complex. The resulting ZCS‐SMSI‐120/PV exhibits a hydrogen evolution rate of 48.7  mmol·g^−1^·h^−1^ without the use of noble metals, representing one of the highest values reported to date. This direct SMSI synthesis approach holds promise for applications in H_2_ production, CO_2_ reduction, biomass conversion, organic synthesis, and so on.

## Graphical abstract

A photobreeding strategy enables the room‐temperature construction of strong metal–support interactions (SMSI) on Zn_0.8_Cd_0.2_S, achieving both direct SMSI formation and continuous interfacial tuning. UV‐induced photoreduction of Zn^2+^ generates metallic Zn, which is subsequently encapsulated by CdS, offering tunable and enhanced catalytic performance.

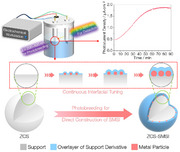

## 1 Introduction

Loaded catalysts with well‐defined metal–support structures are indispensable in modern industrial applications,^[^ ^1^ ^]^ particularly in heterogeneous catalysis for energy conversion, pollutant removal, and fine chemical synthesis.^[^ ^2^ ^]^ The interaction between metal nanoparticles and the support material significantly influences catalytic performance, affecting factors such as activity, selectivity, and stability.^[^ ^3^ ^]^ Since Tauster and colleagues first described in 1978 the suppression of CO and H_2_ chemisorption on platinum‐group‐metal‐loaded reducible oxide‐supported catalysts following high‐temperature reduction treatments,^[^ ^4^ ^]^ the concept of strong metal–support interactions (SMSI) has emerged as a key strategy for optimizing the interfacial properties of metal catalysts. SMSI can not only facilitate efficient electron transfer at the metal–support interface but also induce structural modifications in active sites, thereby enhancing catalytic performance.^[^ ^5^ ^]^ Various strategies have been developed to achieve SMSI, including high‐temperature treatments under oxidative/reductive atmospheres,^[^ ^6^ ^]^ adsorbate‐mediated methods,^[^ ^7^ ^]^ and approaches like wet chemistry^[^ ^8^ ^]^ and atomic layer deposition,^[^ ^9^ ^]^ many of which require elevated temperatures. Recently, mechanochemistry,^[^ ^10^ ^]^ soft‐chemistry,^[^ ^11^ ^]^ and photoinduction^[^ ^12^ ^]^ have been demonstrated as effective mild‐condition approaches to induce SMSI without high‐temperature treatments. Nevertheless, these methods still face challenges in achieving continuous and tunable control over SMSI formation, which is crucial for fine‐tuning the electronic structure properties of the catalyst interface. Such tunability is critical not only for systematically studying the influence of interface structures on catalytic activity and stability, but also for expanding SMSI strategies to a broader range of support materials.^[^ ^10^ ^]^ This in turn facilitates the development of more versatile catalyst design methodologies and promotes a deeper understanding of structure–property relationships across diverse catalytic systems.

**Figure 1:**
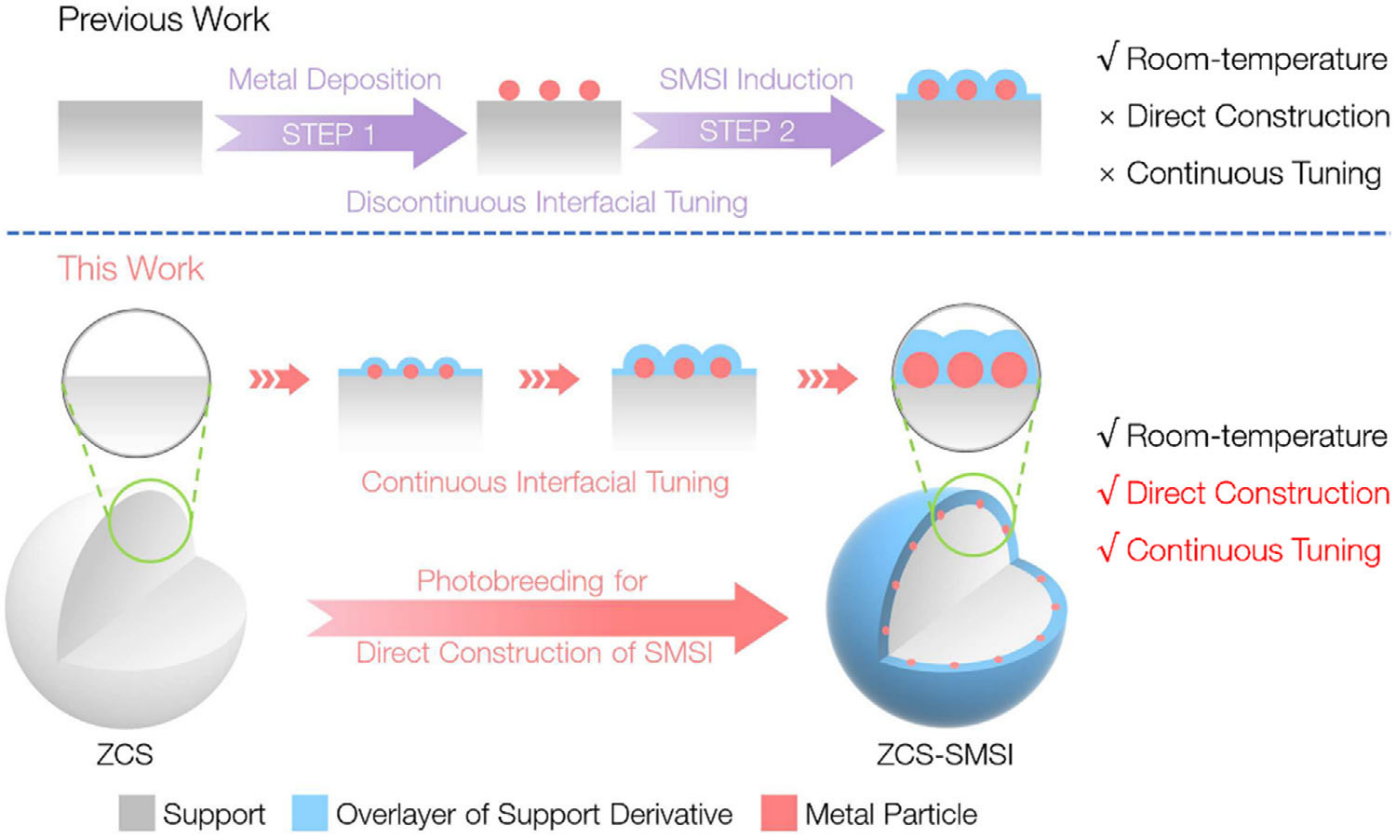
Comparison between conventional and photobreeding strategy for constructing strong metal–support interactions (SMSI).

In this work, a photobreeding method (the direct growth of metal nanoparticles from the parent material through a photochemical reaction at room temperature, without external species—similar to seeds germinating and growing directly from soil)^[^ ^13^ ^]^ is employed to enable the direct synthesis of SMSI catalysts on Zn_0.8_Cd_0.2_S at room temperature without requiring additional treatments. This approach leverages the UV‐induced photochromic reaction of ZnS to generate metallic Zn in situ,^[^ ^13^ ^]^ while the non‐photochromic CdS component encapsulates the formed Zn, thereby establishing strong metal–support interactions (**Figure** **1**). Unlike conventional SMSI formation methods, photobreeding offers precise and continuous tuning of interfacial properties by simply adjusting the irradiation duration, providing a controllable approach for catalyst optimization. Furthermore, this method not only simplifies the synthesis process but also minimizes energy consumption, offering a promising strategy for designing next‐generation SMSI catalysts. The as‐prepared SMSI catalyst on Zn_0.8_Cd_0.2_S demonstrates a 3.1‐fold enhancement in catalytic performance compared to the initial catalyst, highlighting the effectiveness of the photobreeding approach for achieving SMSI and facilitating efficient electron transfer at the interface. To further boost performance, viologen—a well‐known electron mediator—was introduced to form a surface charge transfer complex. The resulting ZCS‐SMSI‐120/PV catalyst achieves a hydrogen evolution rate of 48.7  mmol·g^−1^·h^−1^ under UV–vis light and an apparent quantum yield of 3.0% at 350 nm without the use of noble metals, ranking among the highest values reported for photocatalytic hydrogen production. This work demonstrates the potential of photobreeding as a general, energy‐efficient, and tunable strategy for designing SMSI catalysts with tailored interfacial properties.

## 2 Results and Discussion

### 2.1 Synthesis of Catalyst and Identification of SMSI Effect

**Figure 2:**
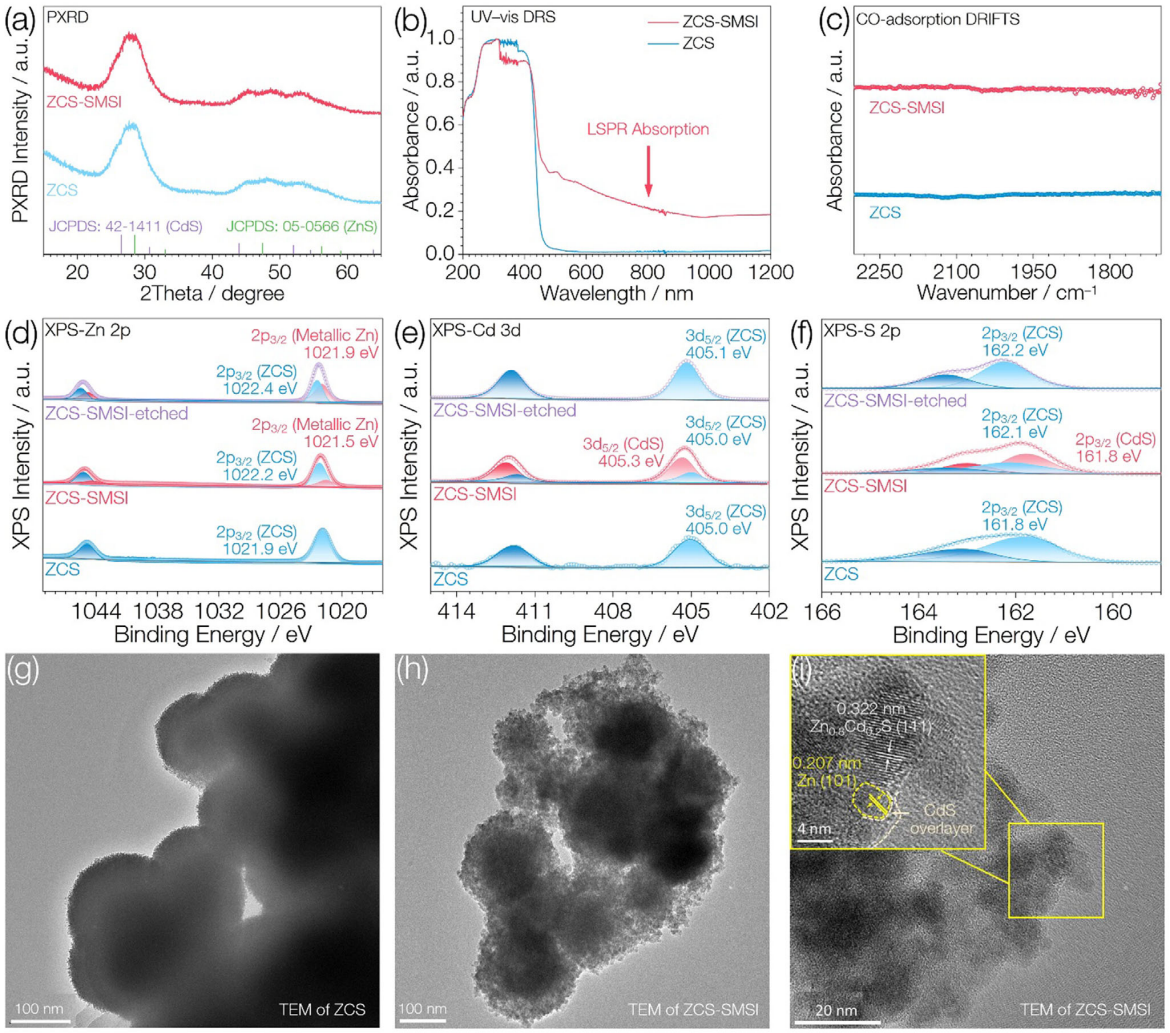
Phase characterization of catalysts. a,b) PXRD, UV–vis DRS of ZCS and ZCS‐SMSI. c) CO diffuse reflectance Fourier transform infrared spectroscopy of ZCS‐SMSI and ZCS/Pt. d–f) High‐resolution Zn 2p, Cd 3d, and S 2p XPS spectra of ZCS, ZCS‐SMSI, and etched ZCS‐SMSI (etching was used to remove the CdS overlayer). g,h) TEM images of ZCS and ZCS‐SMSI. i) HRTEM image of ZCS‐SMSI.

Zn_0.8_Cd_0.2_S (ZCS) was synthesized via a modified solvothermal method based on a previously reported procedure,^[^ ^14^ ^]^ using Zn(OAc)_2_·2H_2_O, Cd(OAc)_2_·2H_2_O, and SC(NH_2_)_2_ in ethylene glycol, with a Zn‐to‐Cd molar ratio of 4:1. The reaction yielded a yellow powder upon completion. The powder X‐ray diffraction (PXRD) pattern of this yellow powder reveals the crystalline phases of cubic ZnS (JCPDS No. 05–0566) and CdS (JCPDS No. 42–1411). Notably, the diffraction peaks are shifted relative to the standard JCPDS data (**Figure** **2**a), which confirms the formation of a solid solution.^[^ ^14^ ^]^ Transmission electron microscopy (TEM; Figure 2g) images show that the ZCS solid solution consists of nanospheres with an average diameter of ≈ 200 nm. Energy‐dispersive X‐ray spectroscopy (EDS) analysis reveals a uniform distribution of Zn, Cd, and S elements, further supporting the successful synthesis of the ZCS solid solution (Figure S1, Supporting Information). X‐ray photoelectron spectroscopy (XPS) analysis indicates that the main constituent elements of the ZCS solid solution are Zn, Cd, S, and O (Figure S2, Supporting Information), with O primarily originating from surface OH species (Note S1, Supporting Information).^[^ ^13^ ^]^ The UV–vis diffuse reflectance spectrum (DRS) shows that the maximum absorption peak and absorption edge of the ZCS solid solution appear at ≈ 320 and 480 nm, respectively (Figure 2b).

After UV (*λ* = 200–400 nm) irradiation, the yellow powder turned brown. UV–vis DRS data (Figure 2b) show that this brown powder retains the original maximum absorption peak of ZCS at 320 nm, while exhibiting a broad absorption band extending beyond 1200 nm, characteristic of the localized surface plasmon resonance (LSPR) effect of metal nanoparticles. The LSPR absorption band is attributed to nanometallic Zn (Note S2, Supporting Information), supported by the observed metallic Zn signals in Zn 2p XPS spectra (Figure 2d) and the known photochromic behavior of ZnS,^[^ ^13^ ^]^ along with the confirmed inertness of CdS (Figure S3, Supporting Information). Furthermore, the reversible color change of ZCS under UV light and dark storage (Figure S4, Supporting Information) provides additional evidence for the reversible photoreduction of Zn(II) to Zn(0), consistent with the XPS results and previous reports on ZnS. XPS data also indicate the presence of CdS in the brown powder (Figure 2e,f). The PXRD pattern of the brown powder remains nearly identical to that of ZCS (Figure 2a), indicating that the generated metallic Zn and CdS are either poorly crystalline or present in a low concentration. For the brown powder, the binding energy of Cd 3d_5/2_ for the ZCS component is nearly identical to that of the initial ZCS, while that of the CdS component is close to the standard value of CdS^[^ ^15^ ^]^ (405.3 eV, Figure 2e). In contrast, the Zn 2p_3/2_ binding energy of the ZCS component is shifted by +0.3 eV relative to the initial ZCS, whereas the metallic Zn component shows a shift of −0.6 eV compared to standard metallic Zn^[^ ^15^ ^]^ (1022.1 eV, Figure 2d). Similarly, the S 2p_3/2_ binding energy of the ZCS component is shifted by +0.3 eV relative to the initial ZCS, and that of the CdS component is shifted by +0.4 eV relative to standard CdS^[^ ^16^ ^]^ (161.4 eV, Figure 2f). After etching, the Zn, Cd, and S binding energies associated with the ZCS component remain essentially unchanged compared to the pre‐etching state (Figure S5, Supporting Information), and the electron‐donating CdS component disappears (Figure 2e,f), while the signal intensity of metallic Zn increases significantly compared to that before etching (Figure 2d). These results indicate that CdS, a derivative of ZCS acting as the support, encapsulates the metallic Zn, and that strong electron transfer occurs from both the support and CdS to the metallic Zn (Note S3, Supporting Information), suggesting the formation of SMSI and successful synthesis of ZCS‐SMSI. The TEM image shows that ZCS‐SMSI features fragmented nanosphere edges (Figure 2h). Metal Zn nanoparticles are located at the edge regions of the ZCS support and are encapsulated by CdS, as evidenced by lattice fringes of 0.207 and 0.322 nm, which correspond to the (101) of metallic Zn and the (111) of ZCS, respectively (Figure 2i). These structural features provide direct evidence of the encapsulation of metal Zn by the support‐derived CdS, further supporting the formation of an SMSI.^[^ ^3^ ^]^ CO diffuse reflectance Fourier transform infrared spectroscopy (CO‐DRIFTS) shows no observable peak related to CO adsorption on ZCS, and likewise, ZCS‐SMSI obtained via the photobreeding method with metallic Zn generation also shows no detectable peak (Figure 2c). These results suggest that CO adsorption on Zn nanoparticles in ZCS‐SMSI is strongly suppressed due to encapsulation by the CdS layer, providing further evidence for the formation of SMSI.^[^ ^5^ ^]^ The EELS mapping (Figure S6, Supporting Information) reveals that the metallic Zn nanoparticles are encapsulated by CdS, providing compelling evidence for the presence of SMSI.

### 2.2 Promotional Effect of SMSI on the Charge Separation

**Figure 3:**
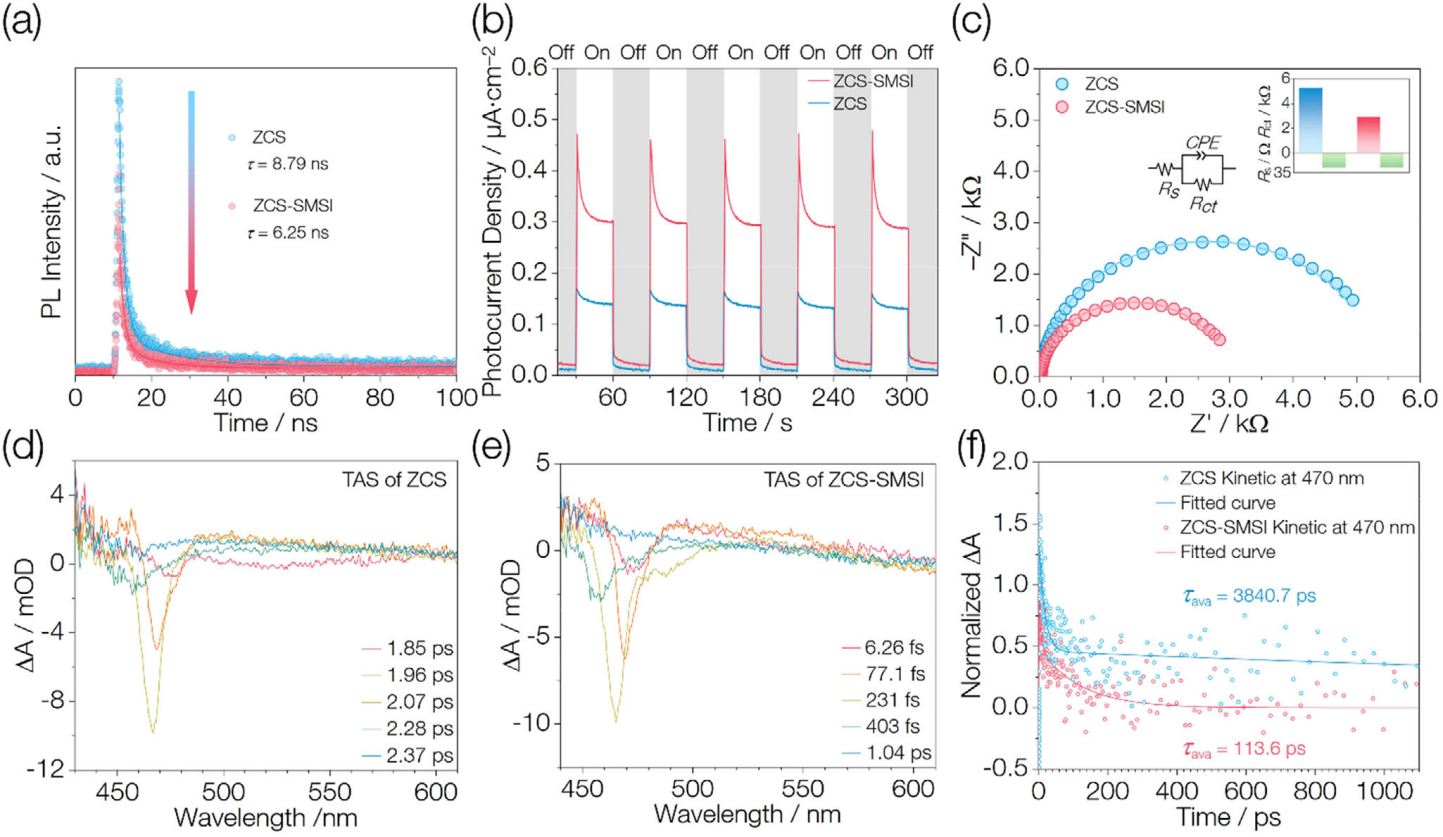
Optical and photoelectrochemical properties of catalysts. a) Time‐resolved photoluminescence (PL) spectra of ZCS and ZCS‐SMSI. b) Transient photocurrent responses of ZCS and ZCS‐SMSI under UV–vis light (*λ* > 320 nm). c) Electrochemical impedance spectroscopy (EIS) Nyquist plots of ZCS and ZCS‐SMSI. Inset (left): Equivalent circuit model of the catalyst–electrolyte interface. *R*_s_ is the electrolyte resistance. *R*_ct_ and CPE are the charge‐transfer resistance from the bulk to the surface of the photocatalyst and the constant phase element, respectively. Inset (right): Extracted *R*_s_ and *R*_ct_ values from the fitted EIS Nyquist plots. d,e) Femtosecond transient absorption spectra (TAS) of ZCS and ZCS‐SMSI on the fs–ps timescales under 400 nm excitation. f) Normalized TAS decay kinetics of ZCS and ZCS‐SMSI probed at 470 nm.

The average photoluminescence (PL) lifetimes of the ZCS and ZCS‐SMSI, measured using a 375 nm laser, are 8.79 and 6.25 ns, respectively (**Figure** **3**a). The pronounced reduction in lifetime for ZCS‐SMSI indicates suppressed charge recombination and enhanced charge separation,^[^ ^17^ ^]^ which can be attributed to SMSI‑driven surface modulation: the heterojunction formed among ZCS, metallic Zn, and CdS at the interface accelerates photogenerated‑electron transfer, resulting in rapid PL quenching. Transient photocurrent measurements reveal that ZCS‑SMSI produces a significantly higher photocurrent than ZCS (Figure 3b), reflecting more efficient separation and transport of photogenerated charges.^[^ ^18^ ^]^ Electrochemical impedance spectroscopy (EIS) Nyquist plots (Figure 3c) show comparable electrolyte resistance (*R*_s_) for both samples, whereas the charge‑transfer resistance (*R*_ct_) of ZCS‑SMSI is markedly lower. These combined optical and electrochemical results demonstrate that SMSI‑enabled surface control effectively diminishes interfacial barriers and greatly improves photogenerated charge‑separation efficiency in ZCS‑SMSI.^[^ ^19^ ^]^

To investigate the dynamics of photogenerated carriers, femtosecond transient absorption spectroscopy (TAS) was performed under 400 nm excitation. Both ZCS and ZCS‐SMSI exhibit a distinct negative signal in the 450–480 nm region (Figure 3d,e). This negative feature is assigned to stimulated emission (SE), as supported by the weak absorption and strong emission peak observed beyond 450 nm in UV–vis DRS (Figure 2b) and PL emission spectrum (Figure S7, Supporting Information), respectively.^[^ ^20^ ^]^ In ZCS, the SE signal emerges at 475 nm at 1.85 ps, reaches a maximum at 2.07 ps, and disappears by 2.37 ps (Figure 3d). The delayed appearance and relatively long lifetime of the SE signal indicate that excitons remain in bound states for extended durations before recombination. This behavior reflects inefficient exciton dissociation and limits interfacial charge separation and electron transfer.^[^ ^21^ ^]^ In contrast, ZCS‐SMSI shows a sharp SE signal at 470 nm appearing within 6.26 fs, peaking at 231 fs, and vanishing by 1.04 ps. The ultrafast onset, short lifetime, and spectral evolution suggest rapid exciton relaxation and depletion of low‐energy states. This implies more efficient carrier separation and faster interfacial processes. To quantitatively evaluate the carrier dynamics, biexponential decay fitting was performed at 470 nm—the wavelength showing maximal SE contrast. The fitted results show that ZCS has a *τ*_1_ of 15.9 ps (65.1%) and a *τ*_2_ of 3870.0 ps (34.9%), yielding an average lifetime of 3840.7 ps. In contrast, ZCS‐SMSI exhibits a *τ*_1_ of 6.0 ps (49.9%) and a *τ*_2_ of 119.0 ps (50.1%), with an average lifetime of 113.6 ps (Table S1, Supporting Information). The *τ*_1_ can be attributed to the fast exciton annihilation or dissociation process at high exciton density, and the *τ*_2_ corresponds to excitons being trapped by the interfacial sites or surface states. The significant shortening of both *τ*_1_ and *τ*_2_ in ZCS‐SMSI suggests that SMSI promotes ultrafast exciton dissociation and effectively suppresses exciton trapping at the interface.^[^ ^21^ ^]^ These findings confirm that SMSI accelerates exciton dissociation into free carriers on the femtosecond to picosecond scale. The rapid decay of SE further demonstrates that SMSI substantially alters the coupling pathways between photoexcited electrons and holes, facilitating fast charge separation and directional electron injection into metallic Zn, CdS, or interfacial trap states.

### 2.3 Continuous Interfacial Tuning of SMSI Catalysts

**Figure 4:**
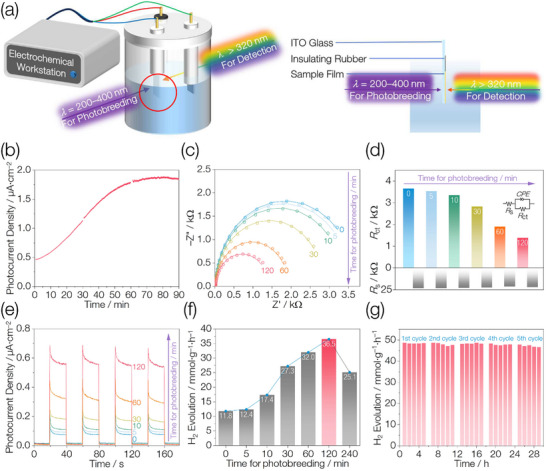
Photoelectrochemical properties and photocatalytic performances. a) Schematic of the dual‐light transient photocurrent measurement setup; the image on the right shows a side view of the red‐circled region in the left image. b) Photocurrent responses of the ZCS‐SMSI measured using the dual‐light setup. Breakpoints in the photocurrent curves result from restarting the test program without turning off the dual‐light source. c) EIS Nyquist plots of ZCS‐SMSI‐x. d) Extracted *R*_s_ and *R*_ct_ values from the fitted EIS Nyquist plots of ZCS‐SMSI‐x. Inset: Equivalent circuit model for the catalyst/electrolyte interface. e) Transient photocurrent responses of ZCS‐SMSI‐x under UV–vis light (*λ* > 320 nm). f) Photocatalytic H_2_ evolution of ZCS‐SMSI‐x under UV–vis light (*λ* > 320 nm). g) Cycling of photocatalytic hydrogen evolution over ZCS‐SMSI‐120/PV under UV–vis light (*λ* > 320 nm).

To demonstrate continuous interfacial tuning of SMSI catalysts via photobreeding, a dual‑light photocurrent setup was constructed (**Figure** **4**a). UV light (200–400 nm) from a UV‑enhanced Xe lamp was directed at the rear side of an ITO glass electrode coated with the sample to induce SMSI in situ, while UV–vis light (> 320 nm) from a solar‑simulated Xe lamp illuminated the front side to generate photocurrent. As shown in Figure 4b, the photocurrent of ZCS‑SMSI increases continuously with longer photobreeding times, eventually reaching a plateau and then slightly declining under extended irradiation. These results demonstrate that photobreeding enables continuous tuning of the SMSI catalyst surface—and hence charge‑separation efficiency—through precise control of UV irradiation duration. ZCS was treated with UV light (200–400 nm) in aqueous suspension for varying durations to obtain a series of ZCS‐SMSI‐x samples, where x denotes the photobreeding time. Time‐resolved PXRD patterns (Figure S8a, Supporting Information) show that all ZCS‐SMSI‐x catalysts display similar diffraction features, indicating that the generated Zn and CdS remain in low content or possess poor crystallinity even with prolonged photobreeding. Time‐resolved UV–vis DRS spectra (Figure S8b, Supporting Information) reveal a gradual enhancement in the intensity of the LSPR band, suggesting an increasing amount of metallic Zn formation with longer photobreeding time. EIS Nyquist plots show that as photobreeding time increases, the arc radius of the ZCS‐SMSI‐x gradually decreases (Figure 4c), corresponding to a reduction in *R*_ct_ (Figure 4d) and thus accelerated interfacial charge separation. These trends are further supported by transient photocurrent measurements (Figure 4e), in which the photocurrent of ZCS‐SMSI‐x increases progressively with longer photobreeding durations, indicating enhanced charge separation efficiency at the interface.

### 2.4 Hydrogen Production Performance of Photocatalysts

The bandgap and the conduction band minimum (CBM) of the ZCS are estimated to be 2.80 eV and −0.772 V (vs Ag/AgCl) according to the Tauc plot and Mott–Schottky curves (Figure S9, Supporting Information), respectively. From a thermodynamic point of view, the ZCS is feasible for H_2_ generation, as its CBM lies above the reduction potential of water (Figure S10, Supporting Information). Under UV–vis irradiation, the initial ZCS catalyst exhibits a hydrogen evolution rate of 11.8  mmol·g^−1^·h^−1^. The photocatalytic hydrogen evolution performance of the ZCS‐SMSI‐x is then evaluated to correlate the surface tuning with catalytic activity. The hydrogen evolution rate is found to increase with photobreeding time, reaching a maximum at 120 min (Note S4, Supporting Information). At this point, ZCS‐SMSI‐120 achieves a rate of 36.5  mmol·g^−1^·h^−1^, representing a 3.1‐fold enhancement compared to pristine ZCS. This pronounced improvement is consistent with the EIS and photocurrent results, which indicate more efficient interfacial charge separation with extended photobreeding duration. Further extension of photobreeding time leads to a decrease in activity, likely due to overconsumption of ZCS, which serves as the source of photoexcited charge carriers. To further enhance photocatalytic performance, viologen^[^ ^22^ ^]^ (a known electron mediator) was introduced to the surface of ZCS‐SMSI‐120 to form a charge‐transfer complex.^[^ ^13^ ^]^ The resulting catalyst, ZCS‐SMSI‐120/PV, exhibits a hydrogen evolution rate of 48.7 mmol·g^−1^·h^−1^ under UV–vis light (Figure 4g) and an apparent quantum yield (AQY) of 3.0% at 350 nm. Notably, this performance was achieved without the use of noble metals, ranking among the highest reported for photocatalytic hydrogen evolution (Table S1, Supporting Information).

Cycling experiments confirm that ZCS‐SMSI‐120/PV maintains stable performance over 29 h of continuous operation (Figure 4g). Furthermore, the PXRD patterns and TEM images before and after cycling remain nearly identical (Figure S11, Supporting Information), demonstrating excellent structural and catalytic stability.

## 3 Conclusion

In summary, this study utilizes a photobreeding strategy that enables the construction and continuous tuning of strong metal–support interactions (SMSI) at room temperature. By utilizing the photochromic behavior of ZnS and the non‐photochromic nature of CdS within a Zn_0.8_Cd_0.2_S solid solution, metallic Zn forms under UV irradiation and becomes spontaneously encapsulated by CdS, resulting in the emergence of SMSI. This work achieves, for the first time, continuously tunable interfacial SMSI through a light‐driven approach. The optimized photocatalyst exhibits a hydrogen evolution rate of 36.5 mmol·g^−1^·h^−1^ under UV–vis light, which represents a 3.1‐fold increase compared to the initial catalyst. To further boost performance, viologen, an electron mediator, is introduced to form a surface charge transfer complex. The resulting ZCS‐SMSI‐120/PV catalyst achieves a hydrogen evolution rate of 48.7  mmol·g^−1^·h^−1^ under UV–vis light and an apparent quantum yield of 3.0% at 350 nm without the use of noble metals, ranking among the highest values reported for photocatalytic hydrogen production. This strategy offers a controllable and energy‐efficient route for interfacial tuning in advanced SMSI‐based catalytic systems.

## Conflict of Interest

The authors declare no conflict of interest.

## Supporting information



Supporting Information

## Data Availability

The data that support the findings of this study are available in the supplementary material of this article.
