# Supplementary material for: Photobreeding Method for Direct Construction and Continuous Tuning of Strong Metal–Support Interactions at Room Temperature
Source: Adv Sci (Weinh). 2025 Sep 9;12(44):e09904. doi: 10.1002/advs.202509904 (PMC12667506; doi:10.1002/advs.202509904)
Supplement: Supplementary file 1 — Supporting Information [file ADVS-12-e09904-s001.pdf]

Supporting information

Photobreeding Method for Direct Construction and Continuous Tuning of Strong Metal–Support Interactions at Room Temperature

Wei-Qiang Huang,[a] Ping Bai,[b] Kai-Bin Jiang*[a,c] and Guo-Cong Guo*[a,c]

[a]State Key Laboratory of Structural Chemistry, Fujian Institute of Research on the Structure of Matter, Chinese Academy of Sciences, Fuzhou, Fujian 350108, P. R. China

[b]Fujian Provincial Key Laboratory of Advanced Inorganic Oxygenated Materials, College of Chemistry, Fuzhou University, Fuzhou, Fujian 350108, P. R. China

[c]Fujian Science & Technology Innovation Laboratory for Optoelectronic Information of China, Fuzhou, Fujian 350108, P. R. China

E-mail: kbjiang@fjirsm.ac.cn; gcguo@fjirsm.ac.cn

List of content

1. Experimental Section S3

2. Tables S6

Table S1. Exponential fits for the fs-TAS decay of ZCS and ZCS-SMSI. S6

Table S2. Comparison of reported photocatalysts for photocatalytic hydrogen production........ S7

3. Graphics S8

Figure S1. EDS images. S8

Figure S2. XPS survey spectra S8

Figure S3. Verification of photochromic inertness of CdS S9

Figure S4. Verification of photochromism of ZCS S9

Figure S5. High-resolution S 2p, Zn 2p and Cd 3d XPS spectra of ZCS and etched ZCS........ S10

Figure S6. The electron energy loss spectroscopy (EELS) mapping of ZCS-SMSI. S10

Figure S7. Photoluminescence (PL) emission spectrum of ZCS........ S11

Figure S8. Phase characterization of ZCS-SMSI-x. S11

Figure S9. Tauc plot and Mott–Schottky curves of ZCS. S12

Figure S10. Band Structure of ZCS........ S12

Figure S11. Catalytic cycle stability. S13

Figure S12. SEM images of ZCS, ZCS-SMSI-120 and ZCS-SMSI-240. .... S13

Figure S13. Photograph of the water droplet on the surface of ZCS........ S13

Figure S14. FTIR spectrum of ZCS. S14

4. Notes S15

Note S1 Discussion on the hydrophilicity and surface-adsorbed species on ZCS. S15

Note S2. Discussion on the broad absorption band related to LSPR........ S15

Note S3. Analysis of electron transfer based on XPS binding energy shifts S16

Note S4. Explanation of performance degradation when photobreeding time extend. S16

1. Experimental Section

Characterization. PXRD patterns of the samples were collected on Rigaku Smartlab X-ray diffractometer with Cu Kα1 radiation (λ = 1.5406 Å). SEM images were recorded on a Zeiss Sigma 300 field emission scanning electron microscope. The used accelerating voltage is 3 kV. Transmission electron microscopy (TEM), high resolution TEM (HRTEM), high angle annular dark field scanning TEM (HAADF-STEM), and elemental mapping were performed on a FEI Talos F200S G2 microscope operated at 200 kV. PL spectra were tested through a FLS 1000 fluorescence spectrophotometer with a Xenon lamp (500 W) and 375 nm laser (EPL-375, maximum average power 5mW, pulse width 63.7 ps). PL emission spectra were measured with the same experimental settings. Time-resolved PL spectra were tested under 375 nm laser excitation using a Red PMT R928P detector. All the electrochemical measurements were conducted in a three-electrode cell connected to an Ivium electrochemical workstation. DRS were recorded on a PerkinElmer Lambda 950 UV/vis/NIR spectrophotometer equipped with an integrating sphere using $BaSO_4$ as the reference. XPS measurements were performed using a Thermo Fisher ESCALAB 250Xi spectrometer, monochromatic Al-Kα radiation (E = 1486.2 eV) was used in combination with Ar ion beam sputter etching to remove the surface coating (tantalum pentoxide was used as a reference for XPS etch depth). All peak energies were referenced to the adventitious C1s, C–C peak at 284.8 eV for calibration purposes. Time-resolved femtosecond transient absorption (fs-TA) spectroscopy experiment was conducted utilizing a femtosecond laser from Spectrophysics and a Helios instrument from Ultrafast Systems. The sample was photoexcited with a 400 nm pump pulse. The fs-TA spectra of the samples were recorded in a probe light range of 430–650 nm. The fs-TA decay kinetics of the samples were conducted at 470 nm and analyzed using double exponential function fitting. The electron energy loss spectroscopy (EELS) mapping was conducted using a field emission transmission electron microscope (JEM-F200, EELS). Water contact angle measurement is carried out on a contact angle meter (Biolin Scientific) using 4 μL of water droplet. Fournier transform infrared (FTIR) spectrum was recorded over the range of 4000–400 cm^{-1} on PerkinElmer Spectrum, FTIR spectrophotometer with pure KBr pellets acts as the baseline.

Materials. All chemicals were obtained from commercial sources and used without further purification. $Zn(CH_3COO)_2 \cdot 2H_2O$ (AR, 99%, Aladdin Industrial Corporation), $Cd(CH_3COO)_2 \cdot 2H_2O$ (AR, 99%, Sinopharm Chemical Reagent Co., Ltd.), NH_2CSNH_2 (AR, Aladdin Industrial Corporation), ethylene glycol

(AR, 99.5%, Sinopharm Chemical Reagent Co., Ltd.), ethanol (AR, 99.5%, Sinopharm Chemical Reagent Co., Ltd.). All aqueous solutions were prepared with Milli Q ultrapure water (18.2 MΩ·cm).

Preparation of photocatalysts. ZCS nanosphere photocatalysts were synthesized via a solvothermal method. Specifically, 3512.16 mg of zinc acetate dihydrate and 1066.12 mg of $Cd(CH_3COO)_2 \cdot 2H_2O$ were weighed into a 250 mL round-bottom flask, followed by the addition of 50 mL of ethylene glycol (EG). The mixture was ultrasonicated until completely dissolved and then stirred for an additional 30 min to obtain a clear solution. Separately, 3044.89 mg of thiourea was dissolved in 50 mL of EG under ultrasonication and stirred for 30 min to obtain another clear solution. The Zn–Cd precursor solution was heated to 180 °C in an oil bath under stirring, and the thiourea solution was rapidly added dropwise using a constant-pressure dropping funnel. After the addition was completed, the reaction was continued for 1.5 h and then allowed to cool naturally to room temperature. The resulting product was collected by filtration, washed three to four times with deionized water and ethanol, and dried at 40 °C for 12 h. A light-yellow solid powder was obtained. Sample for verification of CdS photochromism was synthesized using a similar approach. Photocatalysts of ZCS-SMSI were synthesized in situ with a UV-enhanced Xe lamp (200–400 nm) in aqueous suspensions of ZCS. Unless otherwise specified, photobreeding time was usually 2 hours.

Electrochemical measurements. Working electrode preparation: Dispersed 6 mg of sample in 2 mL ethanol, and then sonicated for 5 minutes. And 90 μL of sample suspension was cast in an area of 1×1 cm^2 on an ITO glass and dried at room temperature to serve as the working electrode. Ag/AgCl electrode and Pt electrode were used as the reference electrode and counter electrode respectively. 0.5 M aqueous solution of Na_2SO_4 (pH = 7) was used as the electrolyte.

Photocatalytic hydrogen evolution. Photocatalytic hydrogen production experiments were conducted in a custom-built flow reactor system.[1] Typically, 20 mg of photocatalyst was ultrasonically dispersed in 80 mL of an aqueous solution containing 0.35 M Na_2S and 0.25 M Na_2SO_3. The suspension was transferred into a 200 mL quartz photoreactor, and the system was purged with argon (50 mL/min) to remove dissolved oxygen and air. Photocatalysis was carried out under UV–visible light ($\lambda > 320$ nm) using a 300 W solar-simulated Xe lamp. A D08-4F flow indicator (Beijing Sevenstar flow Co., LTD, China) was used to maintain a constant argon flow rate during the reaction. The outlet flow rate was calibrated prior to each experiment. Argon was employed as the carrier gas to transport the evolved hydrogen to a gas chromatograph (Shimadzu GC-2014) for quantification. The AQY was measured under the same

photocatalytic reaction conditions as described above, using a 300 W Xenon lamp equipped with a 350 nm ±10 nm bandpass filter as the light source.

AQY calculation. AQY values for ZCS-SMSI-120/PV were evaluated by equation[2]:

$$\mathrm{AQY} = \frac{2\times \text{ the number of evolved } \mathrm{H_2} \text{ molecules}}{\text{the number of incident photons}} = \frac{2N_{\mathrm{H_2}}}{\frac{E\lambda}{hc}} = \frac{2N_{\mathrm{H_2}}}{\frac{P_{\mathrm{light}}\times S_{\mathrm{reactor}}\times t\times\lambda}{S_{\mathrm{detector}}\times hc}} \times 100\%$$

P_{light}: Xenon lamp irradiation power, $S_{detector}$: Optical power meter detector head area (Beijing Perfectlight Technology Co., LTD, China), $S_{reactor}$: Light receiving area of photocatalytic reactor, t: Reaction time, λ: Wavelength of incident light, h: Planck constant, c: Speed of light.

2. Tables

Sample	τ_1 (ps)	A_1 (%)	τ_2 (ps)	A_2 (%)	τ_{ava} (ps)
ZCS	15.9	65.1	3870.2	34.9	3840.7
ZCS-SMSI	6.0	49.9	119.0	50.1	113.6

The average lifetime (τ_{ava}) can be calculated according to the following equation:

$\tau_{ava} = (A_1\tau_1^2+A_2\tau_2^2)/(A_1\tau_1+A_2\tau_2)$

Table S1. Exponential fits for the fs-TAS decay of ZCS and ZCS-SMSI probed at 470 nm under 400 nm photoexcitation.

Modified photocatalysts	Light source	Noble metal	H_2 production rate (mmol·g^{-1}·h^{-1})	Ref.
Ga-doped carbon nitride	300 W Xe lamp AM 1.5	–	99.0	3
Pi-Ho@$C_{3-x}N_4$	300W Xe lamp (λ > 400 nm)	Pt	6.32	4
Cu_CNi_1/TiO_2	300 W Xe lamp (λ > 320 nm)	–	57.8	5
Py-C=N-BT_F-ac COF	300W Xe lamp (λ > 420 nm)	Pt	28.1	6
Ni-B_x/CdS	3 W LED 420 nm	–	13.4	7
SL-MCS/MW NRs	300 W Xe lamp AM 1.5	–	54.4	8
Tp-Py-COF	300W Xe lamp (λ > 420 nm)	–	22.4	9
Cu/Ni-MOL	300W Xe lamp (λ > 400 nm)	–	2.03	10
ZnCdS	300W Xe lamp (λ > 400 nm)	–	30.1	11
CoPOH/MIL	300W Xe lamp (λ > 384 nm)	–	6.60	12
CuSL-CuBr	300W Xe lamp (λ > 420 nm)	–	50.3	13
CNND/CNUN	300 W Xe lamp AM 1.5	Pt	0.58	14
EY-$Cs_2PtSnCl_6$	300W Xe lamp (λ > 400 nm)	Pt	17.6	15
hc-CdS	350W Xe lamp (λ > 320 nm)	–	32.9	16
P-doped Cu−Zn−In−S	300W Xe lamp (λ > 400 nm)	–	12.2	17
ZCS-SMSI-120/PV	300 W Xe lamp (λ > 320 nm)	–	48.7	this work

Table S2. Comparison of reported photocatalysts for photocatalytic hydrogen production and the method in this work.

3. Graphics

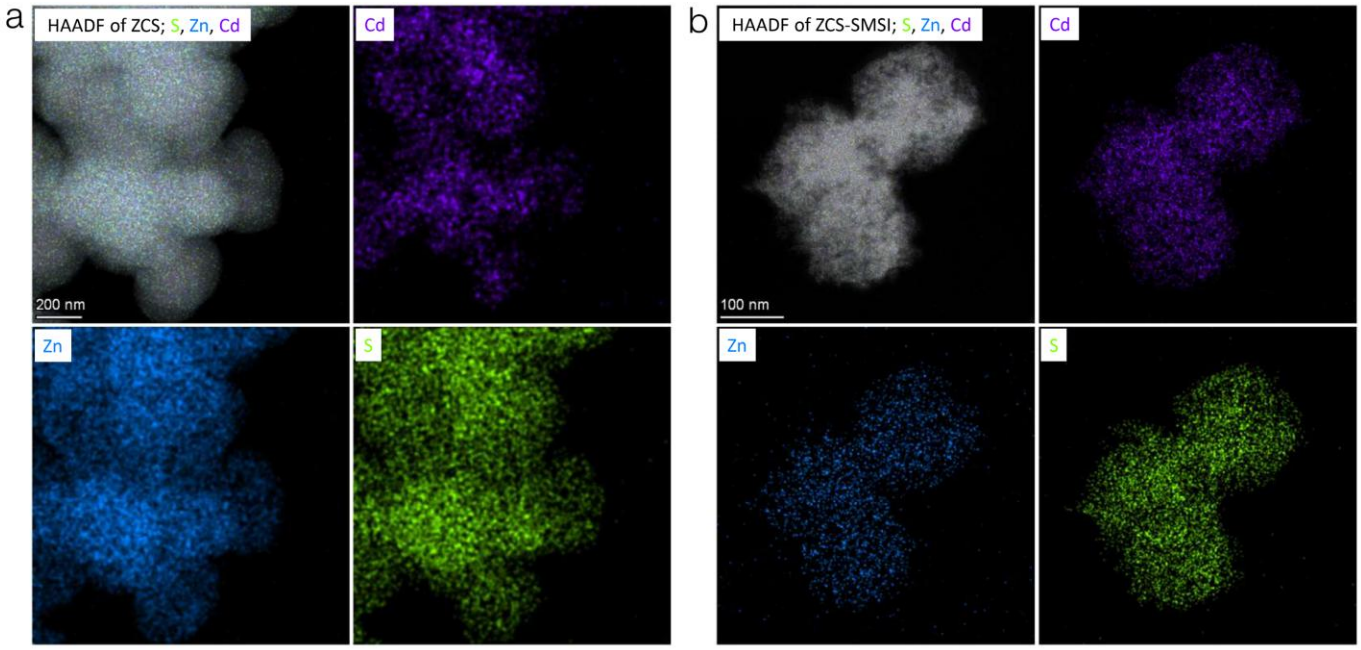

Figure S1. EDS images. (a) ZCS (b) ZCS-SMSI.

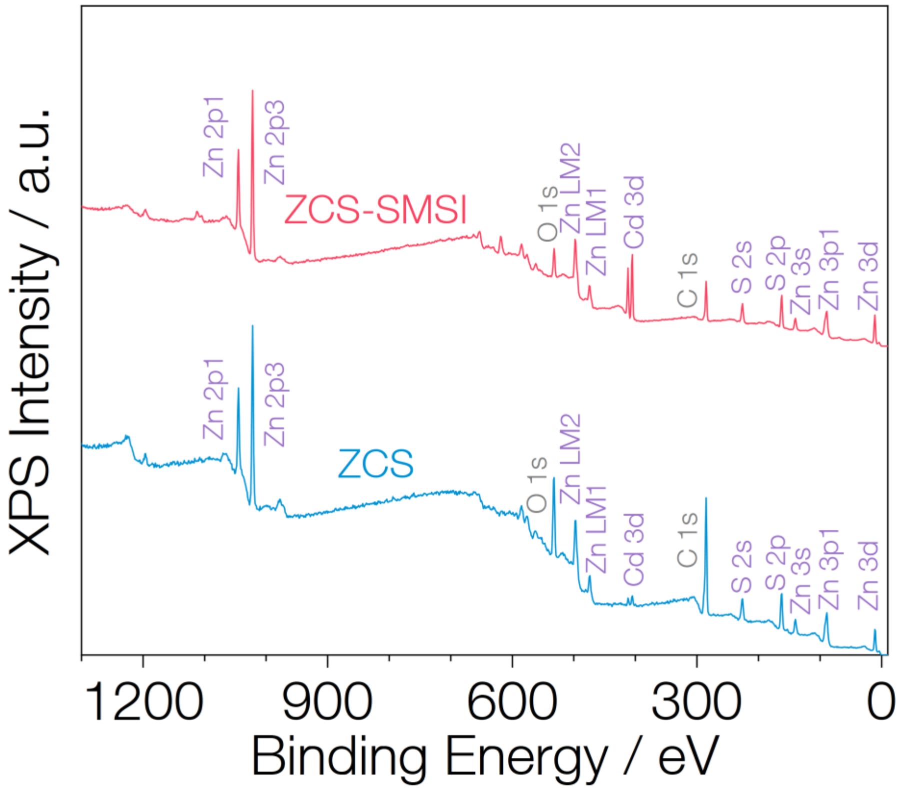

Figure S2. XPS survey spectra of the ZCS, ZCS-SMSI.

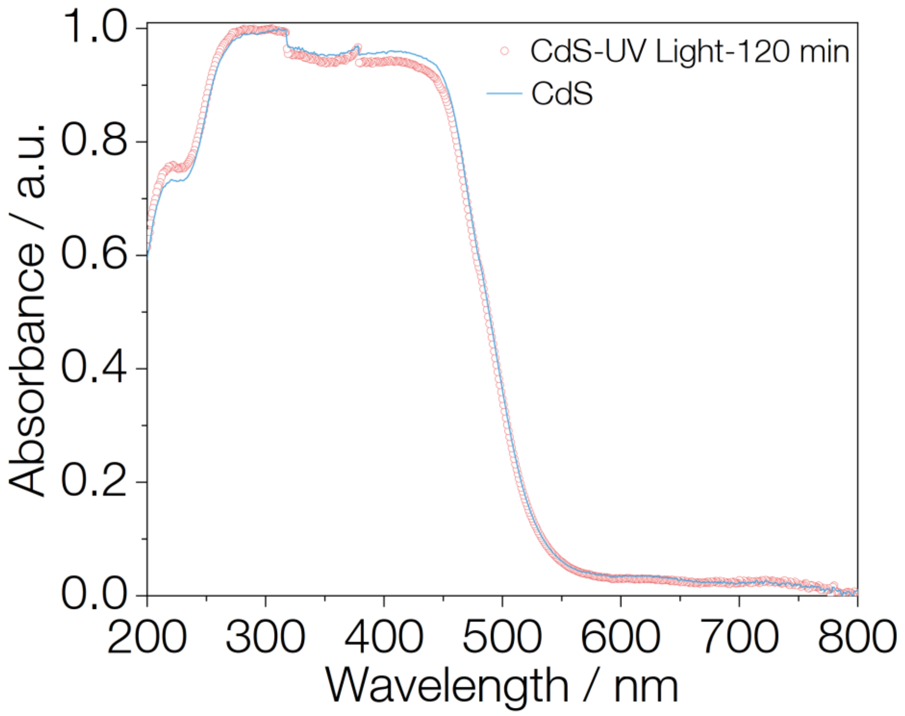

Figure S3. Verification of photochromic inertness of CdS. DRS of CdS before and after UV light irradiation (200–400 nm, 120 min).

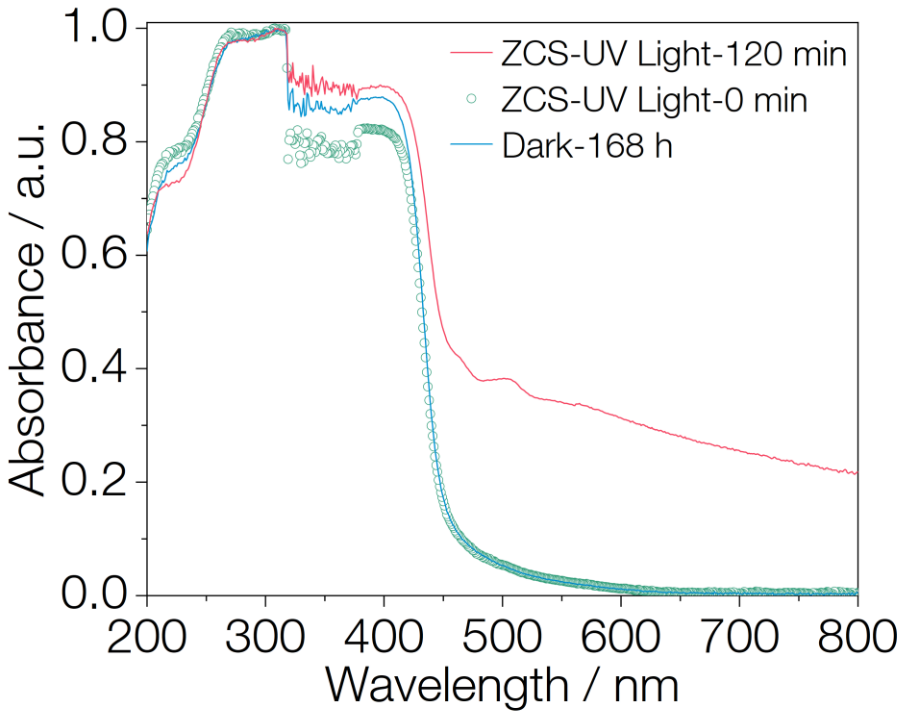

Figure S4. Verification of photochromism of ZCS. DRS of ZCS before and after UV light irradiation (200–400 nm, 120 min) and after subsequent storage in the dark under air for 168 h.

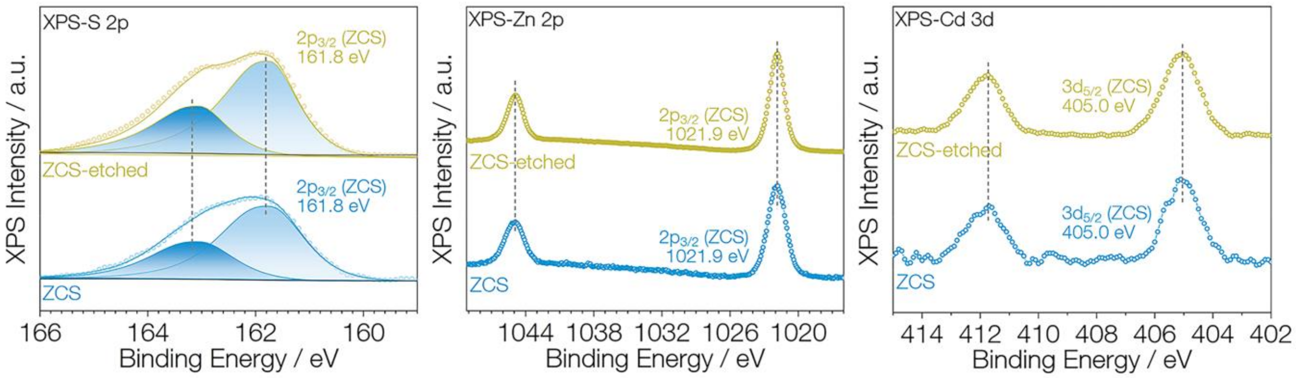

Figure S5. High-resolution S 2p, Zn 2p and Cd 3d XPS spectra of ZCS and etched ZCS.

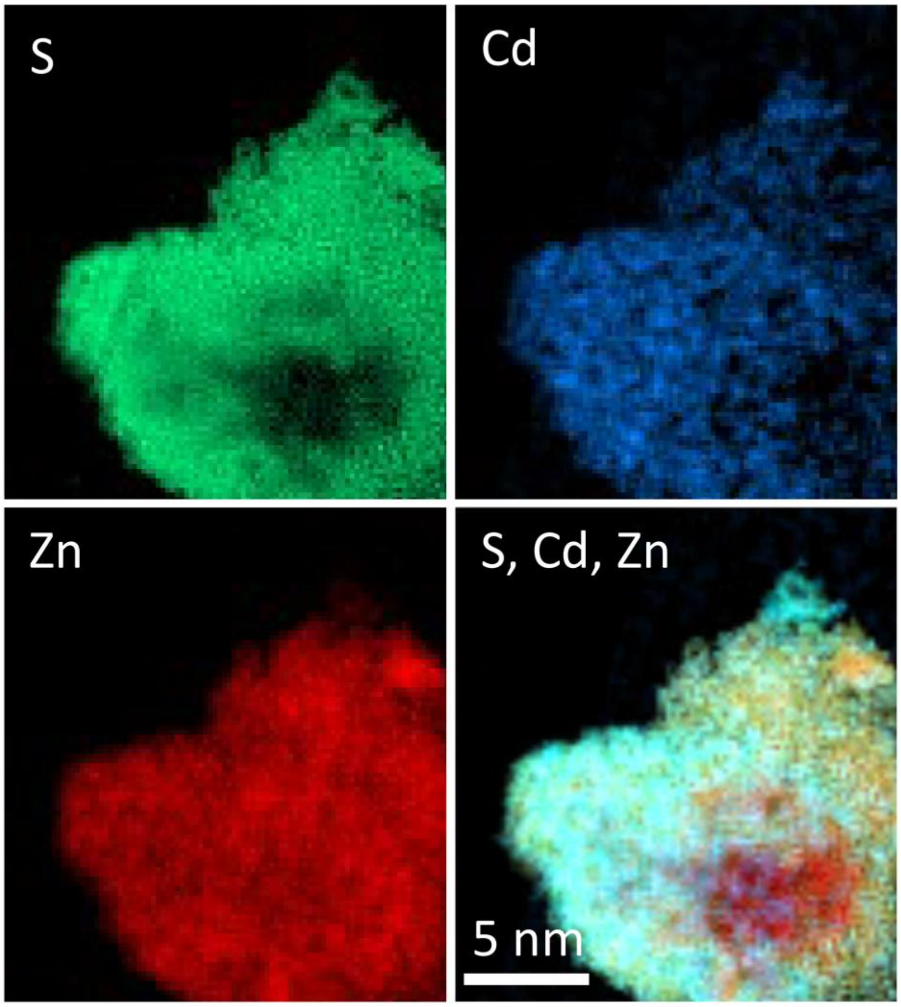

Figure S6. The electron energy loss spectroscopy (EELS) mapping of ZCS-SMSI.

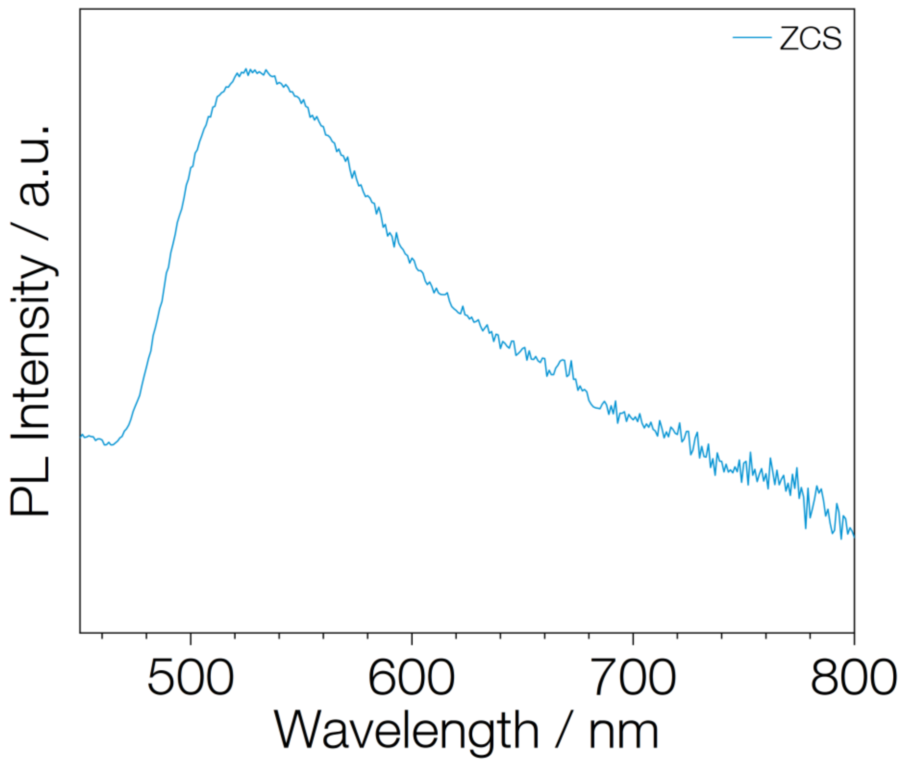

Figure S7. Photoluminescence (PL) emission spectrum of ZCS.

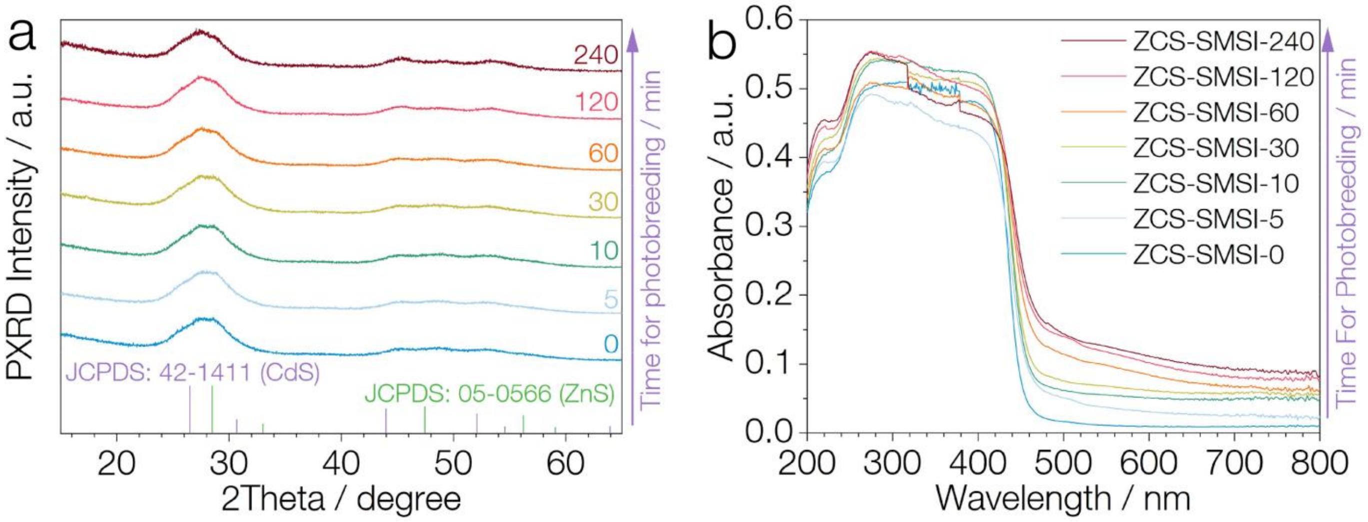

Figure S8. Phase characterization of ZCS-SMSI-x. (a) Photobreeding of ZCS using a UV light (200–400 nm), monitored by time-resolved PXRD. (b) DRS of ZCS upon successive irradiation of UV light (200–400 nm).

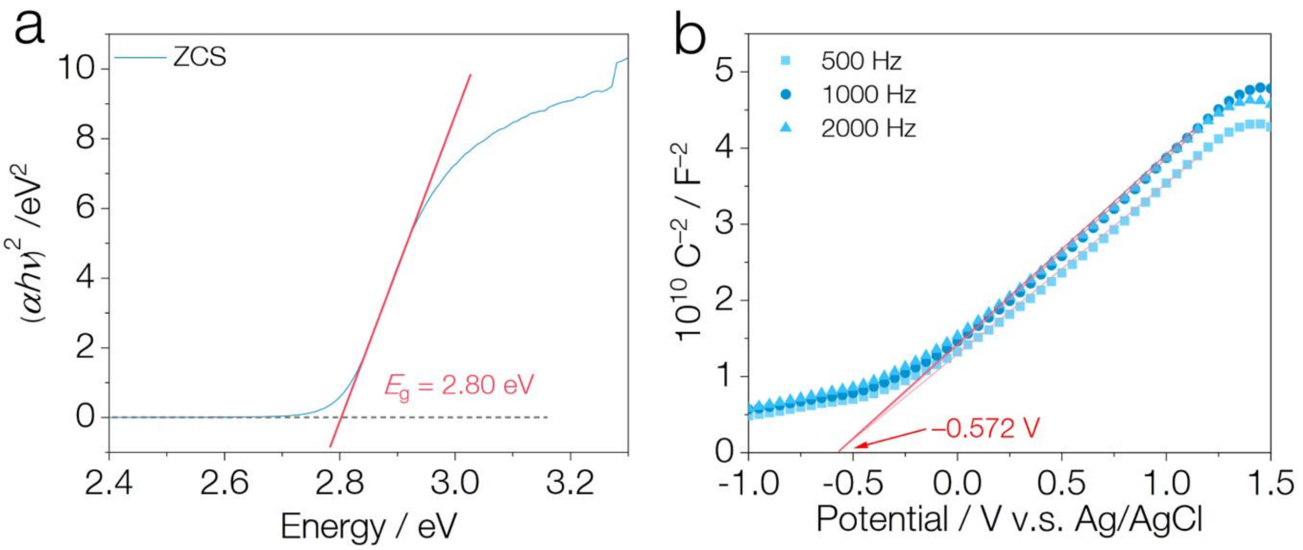

Figure S9. Tauc plot and Mott–Schottky curves of ZCS. (a) Tauc function using a direct transition (exponent = 2) for band gap determination of ZCS[11].(b) Mott–Schottky plots of ZCS.

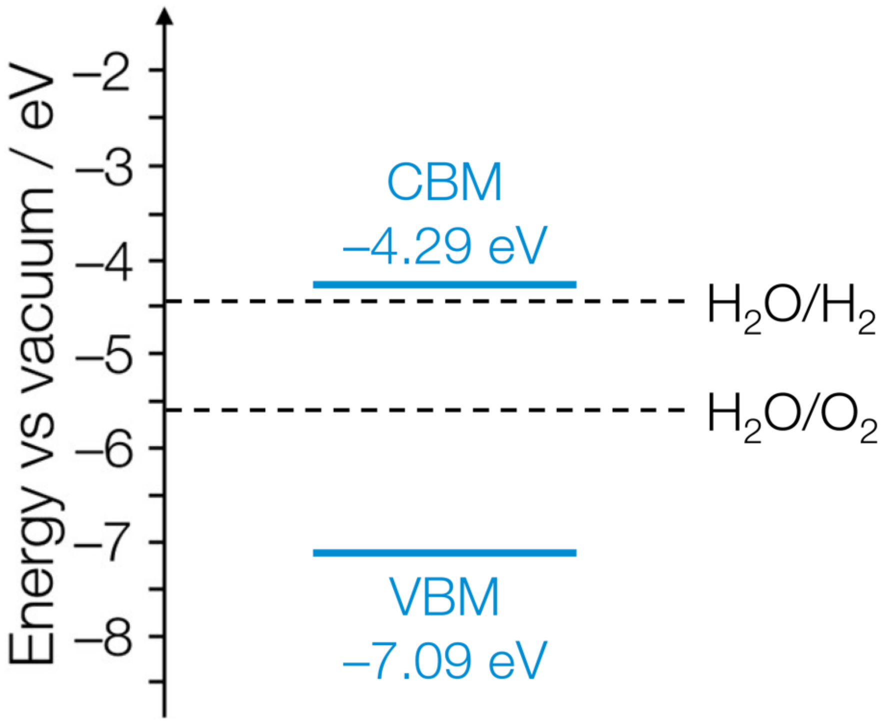

Figure S10. Band Structure of ZCS.

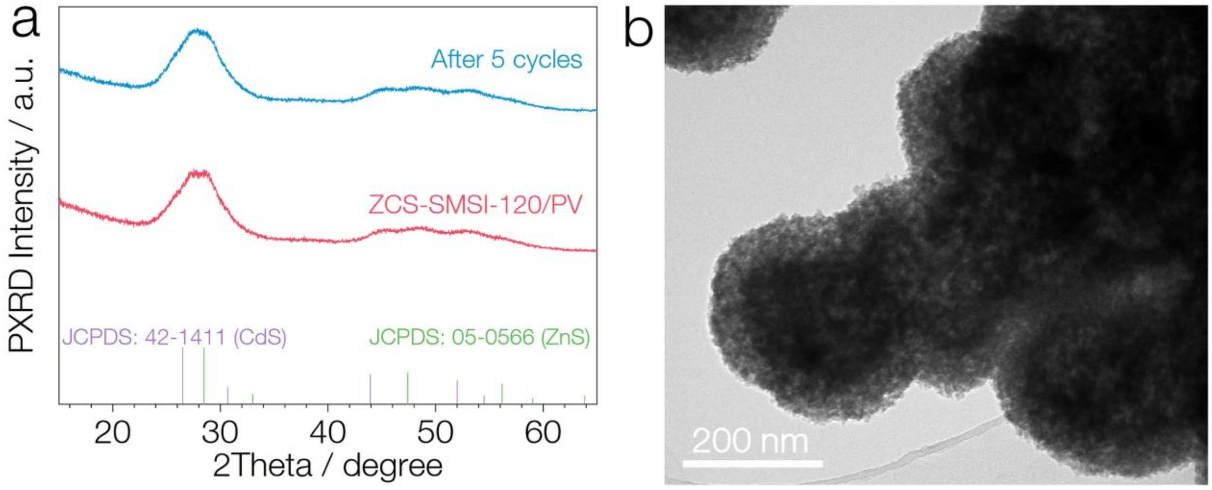

Figure S11. Catalytic cycle stability. (a) Comparison of PXRD patterns of ZCS-SMSI-120/PV before and after 5 cycles of photocatalytic reaction. (b) TEM images of ZCS-SMSI-120/PV after 5 cycles of photocatalytic reaction.

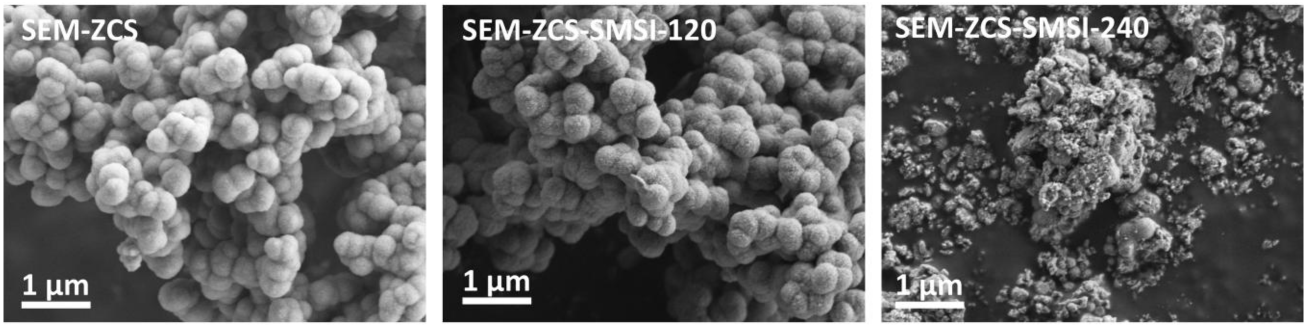

Figure S12. Scanning electron microscope (SEM) images of ZCS, ZCS-SMSI-120 and ZCS-SMSI-240.

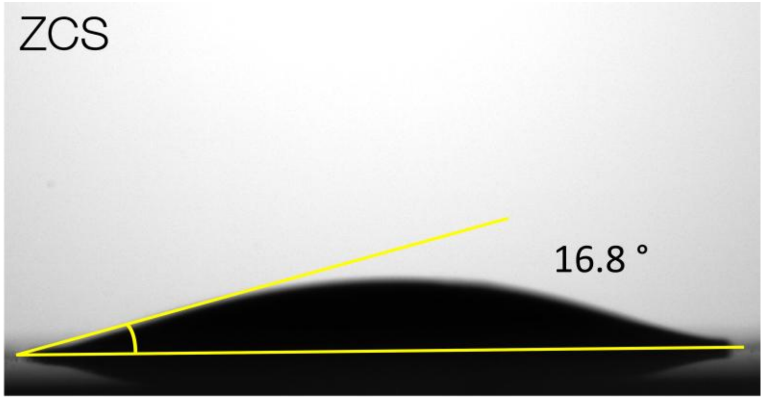

Figure S13. Photograph of the water droplet on the surface of ZCS.

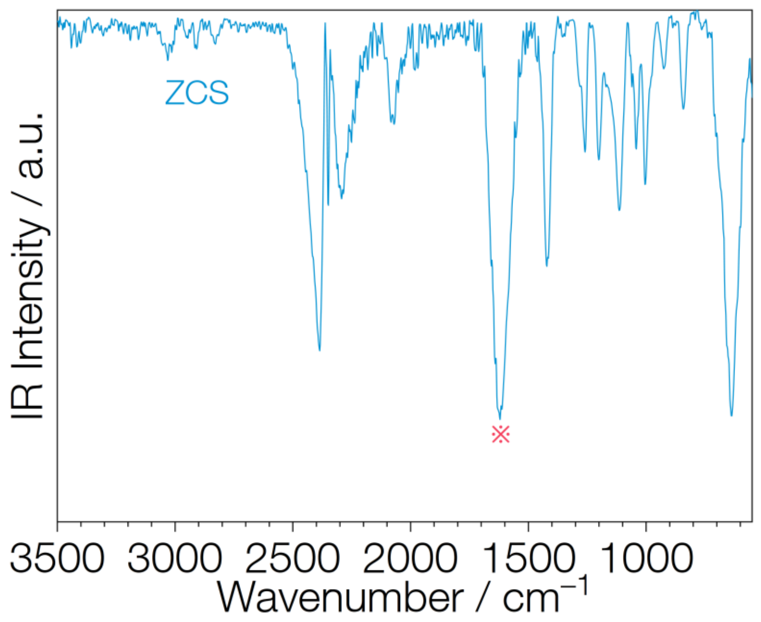

Figure S14. Fourier transform infrared (FTIR) spectrum of ZCS. Absorption peak with red marking is attributed to the absorbed H_2O.

4. Notes

Note S1. Discussion on the hydrophilicity and surface-adsorbed species on ZCS.

The water contact angle of the ZCS sample was measured to be 16.8°, indicating its hydrophilic nature (Figure S13)[27]. Moreover, the FTIR spectrum of ZCS exhibits a characteristic absorption peak at 1620 cm^{-1}, which can be attributed to the bending vibration of adsorbed H_2O molecules, further confirming the presence of water adsorption on the ZCS surface (Figure S14)[28]. These observations are consistent with the XPS analysis, where the relative intensity of the O 1s peak decreases significantly after light irradiation (ZCS-SMSI, Figure S2). This decrease can be assigned to the consumption of surface-adsorbed H_2O during the photochromic reaction, in agreement with previous findings[1]. In addition, since the ZCS sample was synthesized in ethylene glycol, the existence of surface OH species is also reasonable. Taken together, these results consistently demonstrate that ZCS can adsorb water molecules and hydroxyls on its surface.

Note S2. Discussion on the broad absorption band related to LSPR in the DRS spectra of ZCS-SMSI.

It has been reported that metal nanoparticles with sizes of 10–100 nm generally have LSPR effects.[23] The LSPR effect of metallic Zn nanoparticles has been reported.[24][25] The absorption peaks of metal nanoparticles induced by LSPR show broader bands as their sizes and the size inhomogeneity increase in this size range.[24][26]

Due to the low contrast between metallic Zn nanoparticles and the ZCS substrate in TEM images, accurately determining the size distribution of the Zn nanoparticles in our samples is challenging (Figure 2i). However, previous reports on the photobreeding method have shown that the metallic Zn nanoparticles produced tend to be size-inhomogeneous.[1] Such size heterogeneity naturally leads to broad LSPR absorption bands rather than sharp, well-defined peaks. Therefore, the broad absorption feature observed in the UV–vis spectra is consistent with LSPR arising from size-distributed metallic Zn nanoparticles. These factors mutually support the assignment of the broad absorption band to the LSPR effect.

Note S3. Analysis of electron transfer based on XPS binding energy shifts

In ZCS-SMSI, the Zn 2p peak assigned to metallic Zn appears at 1021.5 eV, which is 0.6 eV lower than the standard metallic Zn reference value (1022.1 eV). This downshift indicates electron enrichment of metallic Zn. Meanwhile, the Zn 2p peak associated with ZCS is located at 1022.2 eV, 0.3 eV higher than in pristine ZCS (1021.9 eV), and the S 2p peaks of CdS and ZCS show similar positive shifts (from 161.4 eV to 161.8 eV, and from 161.8 eV to 162.1 eV, respectively). These positive shifts in the binding energy of semiconductor elements (Zn and S) signify electron depletion, consistent with electron donation to metallic Zn. This phenomenon arises from the Fermi level mismatch between semiconductors (ZCS and CdS) and metallic Zn. This electron transfer arises from the Fermi level mismatch at the interface, which drives electrons from the semiconductor to the metal until equilibrium is established. The resulting charge redistribution increases the binding energy of semiconductor elements while decreasing that of the metal. Such behavior is commonly observed in n-type semiconductor–metal systems[18][19], supporting the proposed direction of electron transfer.

After etching, the CdS layer is removed. The Zn 2p peak of metallic Zn in ZCS-SMSI-etched shifts to 1021.9 eV, 0.4 eV higher than in the unetched sample (1021.5 eV), suggesting loss of electron donation from CdS. Meanwhile, the Zn 2p peak of ZCS in ZCS-SMSI-etched increases to 1022.4 eV (from 1022.2 eV), and the Cd 3d and S 2p peaks also shift slightly upward (405.1 eV vs. 405.0 eV; 162.2 eV vs. 162.1 eV), indicating that ZCS now transfers more electrons to Zn to compensate for the absence of CdS. These observations confirm that even after etching, a net electron transfer from ZCS to metallic Zn persists, reinforcing the presence of SMSI at the interface and the dynamic redistribution of interfacial charges based on available donor species.

Note S4. Explanation of performance degradation when photobreeding time is extended beyond 120 minutes.

The ZCS nanospheres subjected to 240 min of photobreeding exhibited severe structural degradation, as revealed by scanning electron microscope (SEM) images showing the collapse of the solid solution framework and its fragmentation into numerous smaller nanoparticles (Figure S11). It is well known that $Cd_{1-x}Zn_xS$ solid solutions possess superior photocatalytic activity compared to individual CdS or ZnS components, owing to their optimized band structures and enhanced charge separation[20][21][22]. However,

once the integrity of the solid solution is compromised—such as through extended photoreduction leading to phase segregation or morphological collapse—the synergistic effects diminish. The structural breakdown not only reduces visible-light absorption but also introduces excessive surface defects and disrupts continuous charge-transport networks, resulting in accelerated recombination of charge carriers.

Moreover, excessive photogenerated metallic Zn nanoparticles are formed during prolonged photobreeding. These metallic Zn particles possess broadband absorption characteristics (Figure S7b), which can compete with the semiconductor for incident photons, thereby decreasing the light-harvesting efficiency of the photocatalyst and further lowering the catalytic performance. In contrast, the sample photobreeding for 120 min retained its overall solid-solution architecture with only moderate surface roughening, thus preserving favorable band alignment and carrier mobility. This structural stability underpins its markedly enhanced photocatalytic performance.

5. References

[1] K. B. Jiang, W. Q. Huang, T. T. Song, P. X. Wu, W. F. Wang, Q. S. Chen, M. S. Wang, G. C. Guo, *Adv. Funct. Mater.* **2023**, *33*, 2304351.

[2] X. Song, G. Wei, J. Sun, C. Peng, J. Yin, X. Zhang, Y. Jiang, H. Fei, *Nat. Catal.* **2020**, *3*, 1027.

[3] W. Jiang, Y. Zhao, X. Zong, H. Nie, L. Niu, L. An, D. Qu, X. Wang, Z. Kang, Z. Sun, *Angew. Chem.Int. Ed.* **2021**, *60*, 6124.

[4] W. Wang, L. Du, R. Xia, R. Liang, T. Zhou, H. K. Lee, Z. Yan, H. Luo, C. Shang, D. L. Phillips, Z. Guo, *Energy Environ. Sci.* **2023**, *16*, 460.

[5] Y. Feng, S. Gong, Y. Wang, C. Ban, X. Qu, J. Ma, Y. Duan, C. Lin, D. Yu, L. Xia, X. Chen, X. Tao, L. Gan, X. Zhou, *Adv. Mater.* **2025**, *37*, e2412965.

[6] Z. Li, B. Cai, Q. Li, D. Zhang, Y. Liang, Y. Liu, Y. Jiao, A. Thomas, X. Zhao, *Angew. Chem.Int. Ed.* **2025**, e202509444.

[7] H. Long, X. Zhang, Z. Zhang, J. Zhang, J. Yu, H. Yu, *Nat. Commun.* **2025**, *16*, 946.

[8] S. Wan, W. Wang, B. Cheng, G. Luo, Q. Shen, J. Yu, J. Zhang, S. Cao, L. Zhang, *Nat. Commun.* **2024**, *15*, 9612.

[9] X. Du, H. Ji, Y. Xu, S. Du, Z. Feng, B. Dong, R. Wang, F. Zhang, *Nat. Commun.* **2025**, *16*, 3024.

[10] Q. P. Huang, C. Yang, Q. Yin, A. A. Zhang, H. X. Liu, L. Li, M. M. Liu, Z. B. Fang, T. F. Liu, *Angew. Chem. Int. Ed.* **2025**, e202502009.

[11] X. Zheng, Y. Song, Q. Gao, J. Lin, J. Zhai, Z. Shao, J. Li, D. Wu, X. Shi, W. Liu, X. Tian, Y. Liu, *Adv. Funct. Mater.* **2025**, 2506159.

[12] Y. Jung, C. W. Lee, B. H. Lee, Y. Yu, J. Moon, H. S. Lee, W. Ko, J. Bok, K. Lee, J. Lee, M. S. Bootharaju, J. Ryu, M. Kim, T. Hyeon, *J. Am. Chem. Soc.* **2025**, *147*, 1740.

[13] Y. J. Cheng, J. Q. Zhao, X. F. Ma, H. L. Zheng, L. He, J. Zhang, Q. Lin, *Adv. Mater.* **2025**, 2503756.

[14] X. Zhu, E. Zhou, X. Tai, H. Zong, J. Yi, Z. Yuan, X. Zhao, P. Huang, H. Xu, Z. Jiang, *Angew. Chem. Int. Ed.* **2025**, *64*, e202425439.

[15] J. Wang, S. He, M. Zhang, F. Yang, Q. Zhang, Z. Li, M. Robert, *Adv. Energy Mater.* **2025**, 2406048.

[16] Y. Zhao, C. Shao, Z. Lin, S. Jiang, S. Song, *Small* **2020**, *16*, 2000944.

[17] L. Wu, F. Su, T. Liu, G. Q. Liu, Y. Li, T. Ma, Y. Wang, C. Zhang, Y. Yang, S. H. Yu, *J. Am. Chem. Soc.* **2022**, *144*, 20620.

[18] Z. Zhang, J. T. Yates, Jr., *Chem. Rev.* **2012**, *112*, 5520.

[19] D. Xu, S. N. Zhang, J. S. Chen, X. H. Li, *Chem. Rev.* **2023**,*123*, 1.

[20] H. Zhou, Q. Liu, W. Liu, J. Ge, M. Lan, C. Wang, J. Geng, P. Wang, *Chem. Asian J.* **2014**, *9*, 811.

[21] A. P. Gaikwad, D. Tyagi, C. A. Betty, R. Sasikala, *Appl. Catal., A* **2016**, *517*, 91.

[22] J. Zhang, S. Yuan, J. Lin, *J. Phys. Chem. C* **2021**, *125*, 25600.

[23] K. B. Mogensen, K. Kneipp, *J. Phys. Chem. C* **2014**, *118*, 28075.

[24] P. K. Kuiri, J. K. Majhi, *Plasmonics* **2016**, *11*, 1233.

[25] X. X. Wang, C. X. Xu, F. F. Qin, Y. J. Liu, A. G. Manohari, D. T. You, W. Liu, F. Chen, Z. L. Shi, Q. N. Cui, *Nanoscale* **2018**, *10*, 17852.

[26] S. Stewart, Q. Wei, Y. Sun, *Chem. Sci.* **2020**, *12*, 1227.

[27] L. L. Liu, C. Cui, F. Chen, J. J. Chen, H. Q. Yu, Y. Xiong, *Angew. Chem. Int. Ed.* **2025**, *64*, e202508718.

[28] X. Guo, L. Liu, J. Wu, J. Fan, Y. Wu, *RSC Adv.* **2018**, *8*, 4214.
